# Antidiabetic Properties of Histamine H1 Receptor Antagonists: A Systematic Review

**DOI:** 10.3390/antiox15091098

**Published:** 2026-08-31

**Authors:** Michał Radzikowski, Mateusz Maciejczyk

**Affiliations:** 1 Students’ Scientific Club “Biochemistry of Lifestyle Diseases” at the Department of Hygiene, Epidemiology and Environmental Health, Medical University of Bialystok, 2C Mickiewicza Street, 15-022 Bialystok, Poland; m.radzikowski20@gmail.com; 2 Department of Hygiene, Epidemiology and Environmental Health, Medical University of Bialystok, 2C Mickiewicza Street, 15-022 Bialystok, Poland

**Keywords:** antihistamines, allergy, diabetes, glycation, oxidation, inflammation

## Abstract

Allergies and diabetes mellitus are highly prevalent, frequently co-occurring diseases. Antihistamines, the first-line treatment for allergies, block H1 receptors but also exhibit anti-inflammatory and antioxidant properties that may improve glucose metabolism. This systematic review examined the evidence for the potential antidiabetic effects of commonly used first- and second-generation antihistamines. A comprehensive search of the PubMed database (1978–May 2026) yielded 17 eligible in vivo, in vitro, clinical, and in silico studies. Most reports indicated that antihistamines improve glucose tolerance, insulin sensitivity, and insulin secretion. Proposed mechanisms include enhanced insulin release, mast cell stabilization, attenuation of immune-mediated responses, reduced nephron alteration, and improved incretin signalling. Although substantial heterogeneity in study designs limits direct comparisons, current evidence suggests a beneficial role of antihistamines in modulating glucose metabolism, particularly for patients managing both allergies and diabetes. These findings highlight the potential for drug repurposing. However, further well-designed clinical trials are required to ensure reproducibility, successful animal-to-human translation, and overall safety before clinical implementation.

## 1. Introduction

Allergies are among the most prevalent diseases in Europe and the United States [1,2,3]. According to the Gell-Coombs classification, allergies are characterized as type I hypersensitivity, defined as an unusual, exaggerated immunological reaction to a specific antigen. Histamine plays a crucial role in allergic reactions. It is a heterocyclic amine derived from imidazole with an ethylamine side chain. Histamine is synthesized from L-histidine with histidine decarboxylase enzyme. Although synthesis is done in many different cells, histamine is primarily stored in granules of mast cells and basophils [4,5]. Histamine undergoes a rather peculiar decomposition pathway; unlike most monoaminergic neurotransmitters, it is not directly degraded by monoamine oxidase-B (MAO-B). Instead, it is primarily methylated by histamine N-methyltransferase (HNMT) to N-methylhistamine, which is subsequently metabolized by MAO-B. Research conducted by Maršavelski et al. [6] revealed that this pre-methylation enhances the substrate’s hydrophobic interactions with MAO-B (rather than HNMT) and significantly stabilizes the enzyme–substrate complex during the rate-limiting step. Furthermore, this specific pathway directs the degradation process to avoid the formation of imidazole acetic acid. Direct oxidation of histamine, which can be catalyzed by diamine oxidase (DAO) in peripheral organs or by MAO-B if HNMT is inhibited, ultimately leads to the production of imidazole acetic acid. This specific metabolite is a γ-aminobutyric acid (GABA) receptor agonist, capable of inducing altered neurochemical and behavioral properties, including excessive sedation. Thus, the mandatory pre-methylation step serves as a specific physiological mechanism to prevent these adverse central nervous system effects [6]. Although histamine is a well-established biological mediator, the exact mechanism of its cellular uptake prior to degradation remains incompletely understood, as a specific histamine transporter has not yet been elucidated. Perdan-Pirkmajer et al. [7] investigated the dynamics of histamine uptake by astrocytes to address this knowledge gap. Their findings indicate that histamine is transported into these cells via at least two distinct pathways. The first mechanism is a highly temperature-dependent, active transport process driven by a sodium ion concentration gradient and ATP. The second mechanism, observed at 4 °C, relies on passive electro-diffusion driven by a concentration gradient. Because histamine is a highly polar molecule and exists predominantly in a protonated state at physiological pH, it cannot freely permeate the lipid bilayer, thereby underscoring the absolute necessity for these carrier-mediated mechanisms [7]. Histamine binds to specific histamine receptors—H1, H2, H3, and H4. Histamine receptors belong to the class A (rhodopsin-like) family of G protein-coupled receptors (GPCRs). They are characterized by a canonical architecture comprising seven transmembrane (TM) α-helices interconnected by three intracellular (ICL) and three extracellular loops (ECL). This forms a barrel-like core that accommodates the orthosteric binding pocket for histamine [8]. A highly conserved aspartic acid residue located within TM3 forms a critical ionic bond with the primary amine of histamine, which is essential for ligand recognition and subsequent receptor activation [8]. Furthermore, a conserved disulfide bridge between TM3 and ECL2 stabilizes the extracellular domain, effectively forming a structural lid over the binding pocket [9,10]. Notably, histamine receptors exhibit significant constitutive activity, maintaining a basal level of signalling independent of ligand binding. The transition to an active conformation is mediated by several highly conserved structural microswitches typical of class A GPCRs, including the DRY, CWxP, and NPxxY motifs [8,9]. In allergies, the H1 receptor (H1R) is of primary importance. It is predominantly expressed on endothelial cells of blood vessels, sensory nerves (especially in the airways), and eosinophils. H1R is coupled to Gq/11 proteins and possesses a binding pocket that is structurally distinct from those of the other histamine receptor subtypes. Specifically, the H1R orthosteric site is noticeably wider and incorporates a substantial hydrophobic cavity, whereas the other receptors feature narrower and more polar binding pockets [11]. Furthermore, H1R signal transduction is primarily mediated by the activation of phospholipase C (PLC) rather than the modulation of cyclic AMP (cAMP) levels, a functional characteristic that differentiates it from the G-coupled histamine H2 receptor (H2R) and the Gi/o-coupled H3 and H4 receptors [8]. When activated by histamine, the H1R can induce mild allergy symptoms such as coughing and rhinitis, as well as more severe effects such as vasodilation (which may lead to hypovolemic shock) and bronchial smooth muscle constriction, potentially causing life-threatening respiratory distress [12]. These complications are among the most dangerous consequences of histamine release. Moreover, excessive H1R activation may lead to increased antigen presentation, upregulated interferon-γ (IFN-γ) and cell adhesion molecules, reduced humoral immunity favouring a Th1 response, enhanced production of inflammatory chemokines, and increased chemotaxis of eosinophils and neutrophils [13]. To counteract these effects, antihistamines are employed.

Antihistamines, also known as H1 blockers (H1B), are a structurally diverse group of drugs that have been used in clinical practice for over 80 years. Despite their initial classification as simple receptor blockers, they are now known to function as inverse agonists of the H1R. This means they not only prevent activation by histamine but also reduce the receptor’s baseline activity, offering additional therapeutic benefits [14]. The voluminous hydrophobic cleft of H1R is of paramount importance for structure-based drug design, as it effortlessly accommodates the bulky diaryl groups of classic antihistamines. By wedging into this cavity, these inverse agonists restrict the conformational mobility of, effectively locking the receptor in an inactive state [11,15]. Two generations of antihistamines have been developed, both including drugs primarily derived from alkylamines, piperazines, and piperidines [16] (Table 1).

Beyond their primary role of H1R inhibition, antihistamines also exhibit anti-inflammatory properties, which are highly valuable in treating allergic diseases [17]. First-generation antihistamines are more lipophilic and readily cross the blood–brain barrier, and their lower selectivity allows for interactions with other central nervous system receptors. For example, hydroxyzine is used to treat insomnia and anxiety, while promethazine has antiemetic properties [18,19,20]. In addition, it was found that apart from the inactivation of H1R function, ketotifen, a first-generation antihistamine, stabilizes mast cell activation [21]. Second-generation antihistamines possess additional hydrophilic groups that serve a dual purpose: physiologically, they restrict blood–brain barrier permeability, while structurally, they facilitate specific interactions with H1R. While the hydrophobic diaryl core anchors deeply within the transmembrane hydrophobic cleft, the extended hydrophilic moiety forms salt bridges with basic residues located in ECL2. This bipartite binding mechanism significantly stabilizes the receptor–ligand complex, thereby increasing both affinity and subtype selectivity [11,15]. Moreover, rupatadine, a second-generation antihistamine, exhibits an antagonist effect on the platelet-activating factor (PAF), which intensifies its antiallergic and anti-inflammatory effects [22]. Nevertheless, the primary use of antihistamines remains in the treatment of allergic conditions, which are often chronic and require long-term therapy. This need supports the continued research into expanded therapeutic uses of antihistamines [23].

Diabetes mellitus (DM) is a chronic disease referred to as epidemic of the 21st century [24]. There are two major types of DM: type 1 (autoimmune or insulin-dependent diabetes), which is characterized by impaired secretion of insulin, the body’s primary hypoglycaemic hormone, due to autoimmune destruction of pancreatic β-cells. Type 2 DM (insulin-resistant diabetes, often associated with lifestyle factors) stems from insulin resistance, which is often triggered by a high and frequent intake of simple carbohydrates. This dietary pattern causes chronically elevated insulin secretion, ultimately reducing the tissues’ sensitivity to it [25]. Despite differing etiologies, both conditions ultimately result in chronic hyperglycaemia. This metabolic state initiates non-enzymatic protein glycation (NEPG), the unregulated binding of monosaccharides to proteins, which subsequently drives the formation of advanced glycation end products (AGEs) [26,27,28,29].

AGEs play a critical role in the pathophysiology of diabetes. Peripheral and renal vascular proteins are particularly susceptible to glycation, which contributes to progressive damage of arteries, capillaries, veins, and nephrons. These changes lead to vascular complications broadly categorized into microangiopathies and macroangiopathies [30]. Microangiopathies affect small vessels and manifest as diabetic retinopathy, nephropathy (potentially progressing to nephrotic syndrome and chronic kidney disease), and neuropathy, which may lead to diabetic foot. In contrast, macroangiopathies involve large arteries, resulting in reduced vascular elasticity and the development of atherosclerosis, coronary artery disease, myocardial infarction, peripheral artery disease, and stroke [30,31]. Circulating serum proteins modified into AGEs bind to their specific receptors (RAGEs), triggering signalling pathways that increase inflammation, notably via nuclear factor kappa B (NF-κB) activation. This disrupts homeostasis by elevating the production of pro-inflammatory cytokines [32]. Protein glycation also plays an important role in the pathogenesis of allergies [33]. Given that DM is more prevalent in people with allergies (1.14% men and 0.96% women vs. 1.06% men and 0.91% women without allergies, crude odds ratio (OR) = 1.06, 95% CI: 0.90–1.24; adjusted for age, socio-economic status, and lifestyle factors OR = 1.25, 95% CI: 1.06–1.49) [34], the use of antihistamines that also exhibit antidiabetic properties could be highly beneficial for patients managing both conditions. Recent studies have explored the potential effects of antihistamines on glucose metabolism, with findings suggesting possible synergistic benefits for patients with both DM and allergies, like enhanced insulin secretion, mast cell stabilization, lowered immunological response, decreased alteration of nephrons, and ameliorated incretin signalling. These investigations have led to the consideration of hypothetical repositioning of antihistamines as antidiabetic agents. This review aims to synthesize the current body of evidence regarding the antidiabetic properties of commonly used H1B, including diphenhydramine, antazoline, promethazine, ketotifen, clemastine, pheniramine, cetirizine, levocetirizine, bilastine, fexofenadine, desloratadine, loratadine, and rupatadine. Currently, antihistamines are routinely prescribed to manage allergies in patients with DM. However, certain medications (particularly first-generation H1B) can stimulate appetite, which is counterproductive for individuals with type 2 DM or concurrent obesity [35].

## 2. Materials and Methods

The literature review involved research articles published between 1978 and May 2026 in the Medline bibliographic database (PubMed). The keyword used in the literature search are listed in Appendix A. The inclusion and exclusion criteria are presented in Table 2.

**Figure 1:**
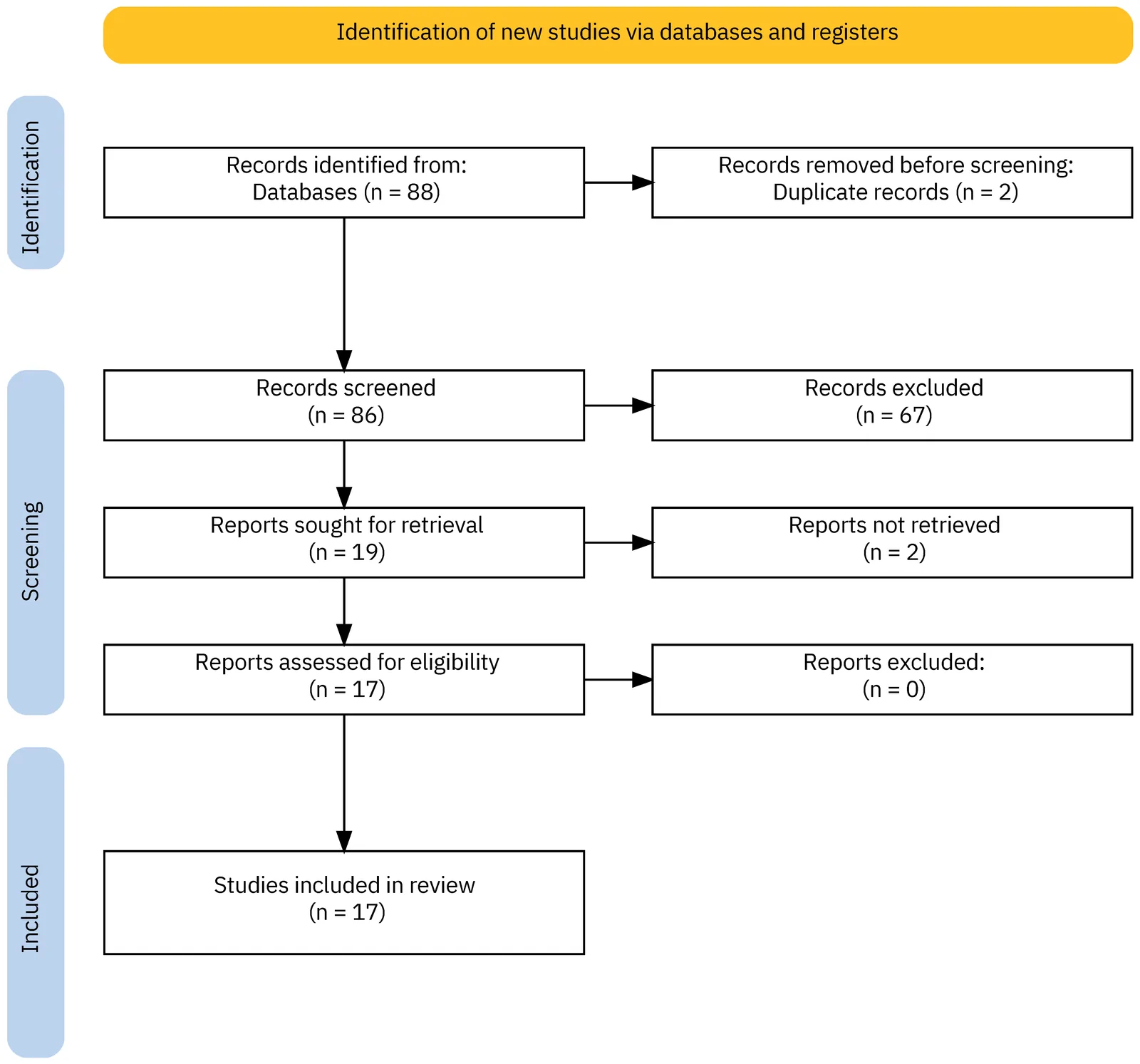
Flowchart of the systematic review process (Prisma).

Two independent researchers performed an initial data analysis based on titles and abstracts of the papers. In the next step, the selected articles were read and assessed for eligibility based on the adopted inclusion/exclusion criteria. Inter-rater reliability was measured with Cohen’s kappa coefficient (κ) (κ = 0.9). To ensure that the collected data meet the highest quality standards, all papers were evaluated for methodology, and the following data were gathered: author names and affiliations, year of publication, research project, sample size, inclusion and exclusion criteria, study duration, and results. The methodology of this systematic review adhered to the Preferred Reporting Items for Systematic Reviews and Meta-Analyses (PRISMA) statement [36]. The completed PRISMA checklist is provided in the Supplementary Material, and the study selection process is summarized in the PRISMA flow diagram [37] (Figure 1). Data from the included studies were extracted independently by two reviewers using a standardized, predefined spreadsheet. Any discrepancies were resolved through discussion and consensus. The following data items were systematically sought and extracted from each article: experimental model details, study design, and endpoints. In instances where specific data points were missing, unclear, or only presented graphically without numerical values in the original reports, no assumptions were made.

Due to the high heterogeneity in study designs, interventions, and outcome measures across the clinical, in vivo, and in vitro studies, a formal meta-analysis was not feasible. Instead, a narrative synthesis of the findings was conducted. The methodological quality and risk of bias of the included studies were appraised independently by two reviewers, with assessment tools strictly tailored to the specific study designs. For human clinical trials, the National Heart, Lung, and Blood Institute (NHLBI) Quality-Assessment Tool for Controlled Intervention Studies [38] was applied to evaluate critical domains such as randomization, blinding, and attrition rates, leading to an overall rating of “Good”, “Fair”, or “Poor”. For in vivo animal intervention studies, the risk of bias was examined across 10 domains using the Systematic Review Centre for Laboratory animal Experimentation (SYRCLE) tool [39], scoring each as “low” (Yes), “high” (No), or “unclear” risk. To evaluate the methodological rigor of the in vitro studies, the recently developed Quality-Assessment Tool For In Vitro Studies (QUIN Tool) [40] was utilized. This tool evaluates 12 specific criteria, including the clarity of objectives, detailed explanation of methodology, outcome measurement methods, and statistical analysis. Each applicable criterion was scored as adequately specified (2 points), inadequately specified (1 point), or not specified (0 points). A final percentage score was calculated to classify the overall risk of bias as low (>70%), medium (50–70%), or high (<50%). Any discrepancies between the primary reviewers during the grading process across all study types were successfully resolved through consensus.

Furthermore, quantitative assessment of reporting bias (e.g., via funnel plots or Egger’s test) was not performed. These statistical methods require a minimum of 10 methodologically homogenous studies per individual outcome inside a meta-analysis to yield adequate statistical power. Because this review synthesizes highly heterogeneous data without a formal meta-analysis, statistical screening for publication bias was deemed mathematically inappropriate; instead, reporting bias was evaluated qualitatively. Similarly, a formal quantitative certainty assessment for each outcome (such as the GRADE system or Summary of Findings tables) was omitted. The majority of the synthesized evidence consists of preclinical in vivo and in vitro studies, for which a universally accepted and standardized GRADE-equivalent system is not fully established. Consequently, the overall certainty of the evidence was appraised qualitatively.

## 3. Results

The systematic review yielded 88 research articles in the Medline database (PubMed). Of those, two were rejected due to duplication of records. Eighty-six abstracts were read, and 19 of those were assessed for eligibility based on the adopted inclusion/exclusion criteria. Two articles were unretrievable. Ultimately, 17 research articles were selected for the study (Table 3).

The three clinical studies evaluating the effects of H1 antagonists in diabetic or prediabetic patients were assessed using the NHLBI Quality-Assessment Tool (Table S1). All three trials [42,48,52] achieved an overall quality rating of “Fair”. While these studies demonstrated comparable baseline characteristics among groups and acceptable adherence/drop-out rates, their methodological scores were primarily limited by small sample sizes, a lack of detailed allocation concealment reporting, and, in some cases, an open-label design. Despite these limitations, the clinical data were deemed sufficiently reliable for qualitative synthesis. The risk of bias for the 12 preclinical animal models was evaluated using the SYRCLE tool (Table S2). The analyzed studies demonstrated a consistently low risk of bias concerning baseline characteristics, incomplete outcome data, and selective reporting. However, a prominent feature of this assessment was a high prevalence of “unclear” ratings in domains such as sequence generation, allocation concealment, and blinding of investigators. It is important to note that an “unclear” rating reflects a historical lack of stringent reporting standards in older preclinical literature rather than inherently flawed experimental designs. Despite these reporting gaps, the metabolic trends and biological responses to H1-receptor antagonism observed across diverse animal models remained highly consistent, strongly supporting the biological plausibility of the findings. The methodological rigor of the two fundamental in vitro studies [55,57] was appraised using the QUIN tool (Table S3). Both studies achieved final scores of 50.0% and 54.5%, respectively, classifying them as having a “Medium” risk of bias. A detailed evaluation revealed that both studies demonstrated excellent clarity in defining their aims, experimental methodologies, comparison groups, and biochemical outcome measurements. The medium-risk classification stems exclusively from a lack of reporting on parameters typically mandated for clinical trials but rarely detailed in basic biochemical research, such as formal sample size calculations, randomization of assay wells, operator calibration, and blinding procedures.

## 4. Discussion

### 4.1. Principal Findings

Of the 17 studies reviewed, 14 demonstrated potential antidiabetic effects of both first- and second-generation antihistamines. Although the observed outcomes were consistent (primarily resulting in improved glucose metabolism, insulin sensitivity, and secretion), the suggested underlying mechanisms varied.

Histamine is a crucial bioactive compound involved in a wide range of physiological processes. Its receptors are expressed not only in mucosal tissues, smooth muscle cells, and vascular endothelium, as previously discussed in the context of allergic reactions, but also in skeletal muscle, the central nervous system, the gastrointestinal tract, and other organs. Precise histamine release is essential for maintaining proper regulation of the sleep–wake cycle, nociception, appetite, insulin sensitivity, and glucose metabolism [58,59,60]. H1R is expressed in pancreatic β-cells, where their activation increases cytoplasmic Ca^2+^ concentration, subsequently stimulating insulin secretion [60]. In skeletal muscle, histamine promotes glucose uptake and utilization, thereby contributing to the reduction of blood glucose levels [61]. However, these effects are observed only under physiological histamine concentrations. Excessive histamine release and consequent overstimulation of H1R lead to dysregulation of glucose metabolism and impaired insulin sensitivity through the induction of oxidative stress [62,63]. Dimlich et al. [43] demonstrated that pharmacological blockade of H1R with diphenhydramine effectively prevents histamine-induced hyperglycaemic shock in rats. Ishiwata et al. [44,45] discovered that adverse effects of some drugs like fluoroquinolones, including impairment of glucose metabolism and excessive histamine release, can be neutralized with diphenhydramine pretreatment in rats. Afzal et al. [46] discovered that diphenhydramine restores impaired glucose tolerance in rats providing valuable insight into the mechanisms linking histamine signalling with glucose homeostasis.

As previously mentioned, first-generation antihistamines, due to their non-specific receptor affinity, not only block H1R but also affect other physiologically important receptors, such as α-adrenergic, dopaminergic, serotonergic, and cholinergic receptors. This non-selectivity can trigger poorly tolerated side effects, including stupor and movement coordination disorders. These, combined with the aforementioned sedation, pose particular risks to patients who must maintain alertness and cognitive function due to their professional or household responsibilities [64,65,66,67]. Consequently, first-generation H1R blockers are generally not preferred for treating chronic allergies. Nevertheless, they have continued to be a subject of research in other pharmacological contexts. Given that insulin secretion is suppressed by α2-receptor stimulation through inhibition of adenylyl cyclase and lowering intracellular cAMP level [68], blockers of this receptor could potentially yield beneficial outcomes for glucose metabolism [69]. Some studies have explored whether first-generation antihistamines might achieve this effect: imidazoline derivatives, known for their α2-antagonist activity, increase insulin release from pancreatic β-cells, not by blocking α2-receptors but rather by inhibiting ATP-sensitive K^+^ channels, which depolarizes β-cells and directly stimulates them to secretion [70,71,72]. Berdeu et al. [41] validated this mechanism in an animal study, where rats treated with antazoline exhibited higher serum insulin levels and a more robust insulin response to glucose administration. Further studies are required to explain the precise mechanisms by which first-generation antihistamines stimulate insulin secretion.

However, multiple factors influence the maintenance of proper glycemia, one of which involves mast cells. These cells release granules containing not only histamine but also a range of pro-inflammatory cytokines and chemokines, such as tumour necrosis factor alpha (TNF-α), interleukin-6 (IL-6), leukotriene C4 (LTC4), and PAF [73,74,75,76]. These mediators alter the cellular redox balance and promote oxidative stress through excessive production of reactive oxygen species (ROS). Elevated ROS levels disrupt cellular membranes and organelles [77]. Although momentary ROS generation may transiently enhance insulin sensitivity, chronic overproduction induces insulin resistance by impairing signalling pathways and receptor function [78,79]. ROS also modulates glucose transporters and glycolytic enzymes, thereby contributing to increased blood glucose levels. Hyperglycaemia itself promotes further ROS generation, establishing a vicious cycle in which elevated glucose levels enhance ROS production, which in turn further stimulates glycolysis and exacerbates hyperglycaemia [80]. In addition, the aforementioned mediators released from mast cells stimulate the formation of reactive nitrogen species (RNS), which exert similar effects to ROS by impairing insulin signalling and glucose transport, ultimately contributing to hyperglycaemia [81,82,83]. Therefore, stabilization of mast cells may prevent oxidative stress and subsequent alterations in glucose metabolism. Chen et al. [47] proposed this mechanism as a potential explanation for the observed efficacy of ketotifen in restoring insulin sensitivity and normalizing glucose metabolism in rats, given its well-established role as a mast cell stabilizer. Similarly, El Haggar et al. [48] observed improvement in fasting blood glucose (FBG) and glycated haemoglobin (HbA1c) levels in their patients after ketotifen administration, which also fits the mentioned actions. Liu et al. [49] conducted an experiment comparing wild-type (WT) mice with *Kit^W-sh/W-sh^* and KitWv/Wv strains, which carry mutations in the c-Kit tyrosine kinase-dependent signalling pathway. These mutations markedly reduce mast cell development and survival, making these models highly suitable for investigations into mast cell-mediated pathophysiology [84]. The authors demonstrated that, when fed an obesity- and diabetes-inducing Western diet, WT mice exhibited significantly greater weight gain and developed pronounced glucose intolerance, whereas these effects were substantially attenuated in *Kit^W-sh/W-sh^* and KitWv/Wv mice. Furthermore, ketotifen treatment improved glucose homeostasis and reduced body weight in WT animals on Western diet, with efficacy comparable to disodium cromoglycate, a well-characterized mast cell stabilizer. These observations strongly support a mechanistic role of mast cells in the development of metabolic dysfunction and suggest that mast cell stabilization may offer therapeutic benefit in diabetes. Returning to the previously mentioned PAF, which promotes redox imbalance by, e.g., activating fibroblast isoform 1 of nicotinamide adenine dinucleotide phosphate (NADPH) oxidase (mitogenic oxidase-1, NOX-1) and inducing TNF-⍺ and IL-6 production [85,86,87], its blockage, which was observed for rupatadine, also brings better glucose metabolism in rats, as Hafez et al.’s [54] study has shown. These studies highlight the importance of proper antioxidant homeostasis, which plays a crucial role in glucose tolerance.

In contrast, second-generation antihistamines are largely free from these side effects, representing a significant advancement for patients with allergies. These drugs are significantly less lipophilic, limiting their penetration through the blood–brain barrier and thus reducing their sedative potential. Additionally, their affinity for H1R has been optimized, minimizing side effects associated with α-adrenergic, dopaminergic, serotonergic, and cholinergic receptors [66]. Moreover, second-generation antihistamines have been found to modulate immune responses and reduce inflammation [17,88], both of which are relevant to allergy sufferers and individuals with diabetes. For instance, levocetirizine has been shown by Shawky et al. [51] to increase insulin sensitivity and secretion, as evidenced by changes in indices such as the oral glucose tolerance test (OGTT) and homeostasis model assessment of β-cell function (HOMA-β) in rats. Levocetirizine also has antioxidant properties, normalizing serum levels of superoxide dismutase (SOD) and malondialdehyde (MDA). Additionally, levocetirizine enhances high-density lipoprotein-cholesterol (HDL-C) levels, which are known to attenuate lipid peroxidation and increase endothelial nitric oxide synthesis, potentially improving aortic contractility. Levocetirizine also acts as an anti-inflammatory agent and modulates glucose metabolism [89,90,91,92,93,94]. This research also indicated that levocetirizine independently exhibits anti-inflammatory properties by reducing serum C-reactive protein (CRP) levels and decreasing liver steatosis. Anbar et al. [50] also investigated levocetirizine’s antidiabetic potential in rats, showing that it helps control blood glucose levels and restore normal HbA1c levels, a key retrospective indicator of DM. This study compared single and combined therapies of levocetirizine with losartan, an angiotensin II receptor blocker used to treat hypertension, often associated with DM due to its nephroprotective properties [95,96,97]. The research demonstrated that this combination successfully reduced or even reversed kidney damage caused by hyperglycaemia, such as renal hypertrophy, polyuria, and proteinuria. Moreover, the glomerular filtration rate (GFR) was nearly normalized, along with serum creatinine and urea levels. The combination therapy also significantly alleviated diabetes-induced focal segmental glomerulosclerosis, interstitial fibrosis, and glomerular basement membrane thickening. Additionally, this treatment lowered levels of redox biomarkers (MDA and SOD) and the profibrotic transforming growth factor beta-1 (TGF-β1) associated with diabetes. Levocetirizine alone also reduced transforming growth factor α (TGF-α) and increased reduced glutathione (GSH) levels. Another observation was the restoration of nitric oxide synthase (NOS) activity, contributing to proper endothelial function. Beyond this, levocetirizine demonstrated protective effects against aortic hypertrophy, a complication of diabetes. Antihistamines, like the previously mentioned levocetirizine and bilastine, have been reported to enhance kidney laboratory results like albuminuria, the albumin-to-creatine ratio, and creatin clearance, hence restoring kidney function disrupted by hyperglycaemia [98,99]. These findings suggest not only the anti-diabetic potential of levocetirizine but also its nephroprotective and aorta-protective properties.

Another target for second-generation H1B was identified by Almasri et al. [100], who discovered that these drugs could inhibit dipeptidyl peptidase-4 (DPP-4), an enzyme that degrades a variety of proteins, including glucagon-like peptide-1 (GLP-1) [101]. GLP-1, produced by intestinal enteroendocrine L-cells, is an incretin that lowers glucose levels by enhancing insulin secretion, increasing β-cell proliferation, and protecting pancreatic islets [102,103]. GLP-1 is rapidly cleaved by DPP-4 under physiological conditions. Due to these properties, GLP-1 analogues (e.g., semaglutide) and DPP-4 inhibitors (e.g., sitagliptin) are successfully used in diabetes treatment [104,105,106]. Through molecular docking studies, it was discovered that fexofenadine binds to DPP-4 similarly to conventional DPP-4 inhibitors. Subsequent in vitro experiments confirmed the inhibitory effect of fexofenadine on DPP-4 and determined its IC50 at 4.6 ± 1.0 µM. Finally, animal studies demonstrated improved glucose metabolism following fexofenadine treatment. Additionally, El-fatary et al. [52] also investigated fexofenadine’s impact on glucose homeostasis in humans and found that it helps with reducing FBG and HbA1c levels.

In addition to the previously discussed diabetes-protective properties, another noteworthy aspect is the antiglycation potential of certain substances. While the majority of currently utilized antidiabetic drugs focus on restoring impaired glucose metabolism and reducing glycemia, they often fail to address the persistently elevated blood glucose levels that contribute to the development of NEPG and its associated complications [107,108]. Consequently, researchers are striving to identify compounds capable of preventing protein glycation and mitigating its detrimental effects. Biedrzycki et al. [57] conducted both in silico and in vitro analyses of several H1-receptor antagonists: H1 blockers from the first (diphenhydramine, antazoline, promethazine, ketotifen, clemastine, pheniramine) and second (cetirizine, levocetirizine, bilastine, fexofenadine, loratadine, desloratadine) generations to explore their potential antiglycation properties. The in silico analysis employed molecular docking techniques, which facilitate the examination of hypothetical affinities and binding sites between specific ligands and receptors. The researchers found that the interactions between H1 blockers and bovine serum albumin (BSA) were moderate to strong, suggesting that these compounds might present antiglycation potential in subsequent in vitro experiments [57]. However, it is important to note that while in silico methods provide valuable theoretical insights, their results are not definitive and should serve as a preliminary guide rather than a conclusive determinant of research direction. The subsequent in vitro analysis aimed to further investigate the antiglycation potential of the selected H1 blockers. Unfortunately, the results did not support the hypothesis of antiglycation activity [57]. Nevertheless, the study also evaluated the antioxidant properties of these compounds, revealing a significant ability to reduce the formation of ROS in the tested models [57]. These findings suggest that while the investigated H1 blockers may not exhibit antiglycation properties or directly mitigate NEPG-related complications, their antioxidant activity could contribute to glycaemic control via an alternative pathway. This highlights the potential for these substances to indirectly influence diabetes management through oxidative stress reduction.

While the aforementioned studies have identified various anti-diabetic properties of certain antihistamines, none of them have been validated in terms of preventing diabetes development. Anvari et al. [56] examined diabetes development in non-obese diabetic mice treated with cetirizine and found no significant impact of this implementation. However, an in vitro study proved promising properties of this compound in preventing cytokine- and hydrogen peroxide-induced β-TC6 cell death [55]. Research by Böhmer et al. [42] aimed to determine whether ketotifen could prevent pancreatic β-cells from autoimmune destruction, and, thus, developing DM in islet cell-antibody-positive prediabetic patients. Prior to administration in humans, these protective properties were confirmed in an animal model using pizotifen, a compound that primarily acts on serotonin receptors, with some H1R activity [109]. The study focused on the vasoconstrictive properties of the compound, as prediabetic insulitis is associated with vascular leakage due to progressive vasodilatation. Unfortunately, Böhmer et al.’s study did not demonstrate protective effects on pancreatic islets, as most patients developed DM within 1 year. The authors highlighted several reasons for the discrepancy between human and animal models, the most significant being that ketotifen administration may have been initiated too late to effectively arrest autoimmune inflammation in the pancreas. Additionally, animal study models are typically highly standardized and strictly controlled, whereas human studies are more complex and difficult to maintain under ideal conditions. The threshold between prediabetic state and early diabetes—where ketotifen is ineffective—is also unclear and difficult to assess based on the immunological and insulin secretion tests available at the time of the study. Despite the lack of confirmation of certain antihistamine properties, this study provided two critical insights:

(1) Although non-human study models are undoubtedly important and play a significant role in scientific development, they should not be considered the definitive direction of research. Instead, scientists should regard them as indicators of probable outcomes for further experiments, which should be tailored and adjusted to conditions specific to the human organism.
(2) Study groups should be selected with maximum precision, taking into account all possible exclusion criteria that may significantly impact the research.

Both allergic diseases and diabetes are chronic conditions that frequently require long-term pharmacotherapy. Therefore, potential long-term metabolic effects and adverse events should be considered when evaluating chronic treatments. Gasheva et al. [53] assessed the consequences of prolonged administration of the H1 receptor antagonist desloratadine in rats. The animals received daily desloratadine for 16 weeks, and the authors report that treated rats developed an obesity-like phenotype and multiple features of metabolic syndrome, including excessive weight gain, increased abdominal/subcutaneous adiposity, hepatic steatosis, elevated fasting blood glucose with relatively normal insulin levels (indicative of insulin resistance), and altered plasma lipid profiles (increased triglycerides and HDL). These alterations were accompanied by mesenteric lymphatic vessel dysfunction (increased lymphatic tone and resistance to flow, and loss of adaptive phasic responses). Gasheva et al. [53] interpret these findings mechanistically: histamine contributes to lymphatic vessel regulation, and chronic H1R blockade may increase lymphatic resistance and impair postprandial lipid transport (e.g., chylomicron handling), thereby promoting mesenteric lipid accumulation and metabolic dysregulation. The authors also position their results within epidemiological observations that regions of United States of America and time periods with higher seasonal allergy rates and antihistamine use overlap with increases in obesity prevalence. However, these population-level data are correlational and do not establish causality. Consequently, while the animal data support a plausible lymphatic-mediated mechanism linking chronic H1R blockade to metabolic disturbances, translation to humans requires cautious interpretation and further prospective studies.

When evaluating the antidiabetic potential of antihistamines, the most critical outcomes are glycaemia, glycated haemoglobin, and the homeostatic model assessments for insulin resistance (HOMA-IR) and HOMA-β, as these parameters provide a direct and comprehensive view of glucose metabolism. Fasting blood glucose and fasting plasma glucose served as baseline measurements across all clinical and in vivo studies included in this review [41,42,43,44,45,46,47,48,49,50,51,52,53,54,56]. Analyzing these parameters allowed for a detailed assessment of glycaemic stabilization, which was shown to improve in the majority of cases. Furthermore, HbA1c reflects the average blood glucose concentration over the preceding 2–3 months, offering greater diagnostic value than point-in-time glycaemia measurements, which can be easily skewed by temporary variables such as acute stress. Notably, HbA1c levels improved in all studies that evaluated this parameter [48,50,52], suggesting that antihistamines exert a prolonged protective effect on glucose metabolism. HOMA indices, which estimate pancreatic β-cell function and peripheral insulin sensitivity, were assessed in two studies. Both reported significant improvements, further highlighting the capacity of antihistamines to restore normal insulin secretion and tissue responsiveness [47,51]. Predictably, in vitro and in silico models did not assess these systemic parameters due to inherent methodological limitations.

**Figure 2:**
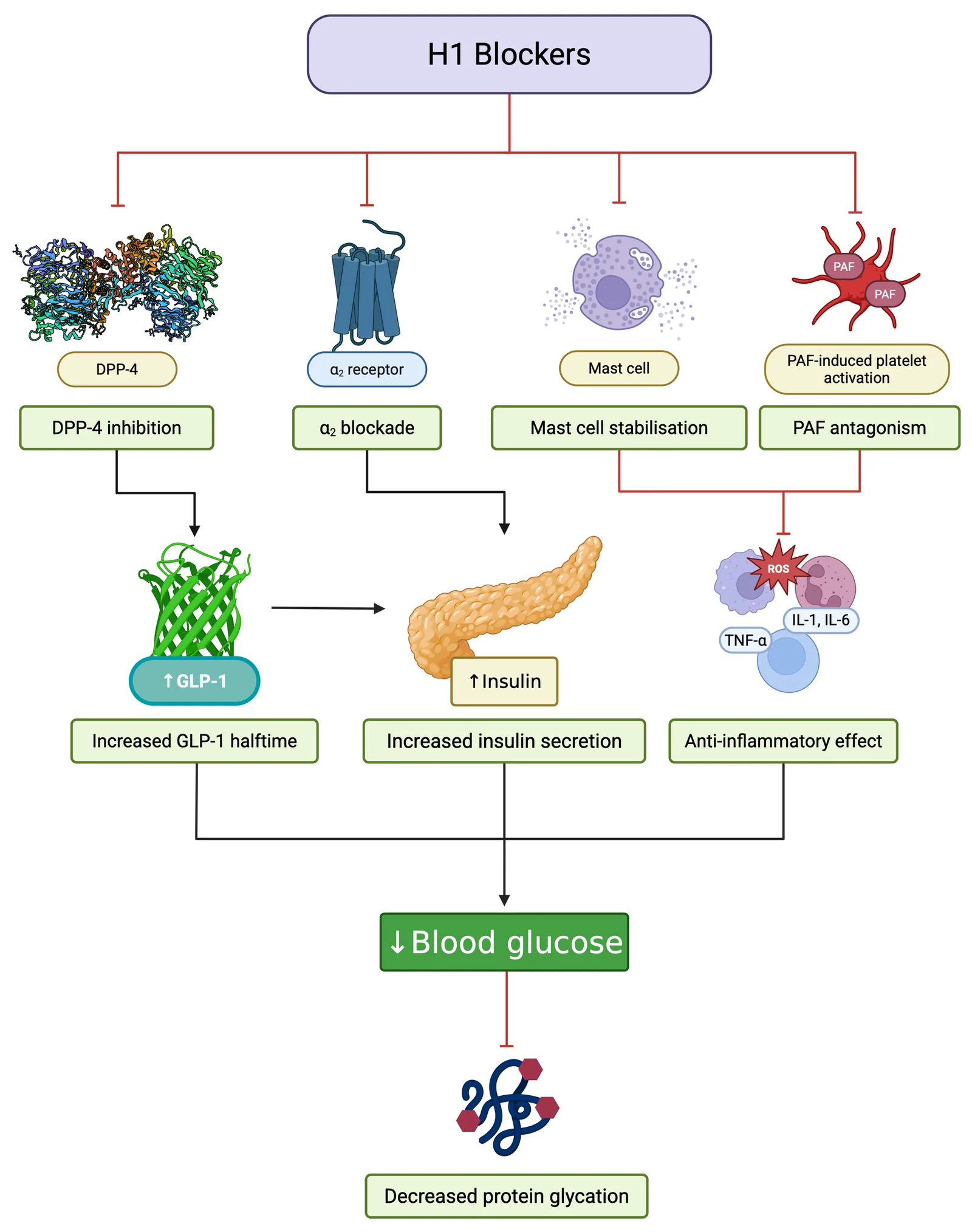
Suggested mechanisms of antidiabetic properties of antihistamines. By preventing mast cell degranulation and platelet activation, H1 blockers decrease inflammation by supressing reactive oxygen species formation and proinflammatory cytokine release. Inhibition of dipeptidyl peptidase-4 leads to increased biological half-life of glucagon-like peptide-1 and, thus, insulin secretion, which is also stimulated by blockade of α2 receptor. Those effects collectively contribute to reduction of glycaemia, and, consequently, protein glycation. Abbreviations: DPP-4, dipeptidyl peptidase-4; GLP-1, glucagon-like peptide-1; IL, interleukin; PAF, platelet-activating faction; ROS, reactive oxygen species; TNF-α, tumour necrosis factor α. Created in BioRender. Maciejczyk, M. (2026) https://BioRender.com/l8ju2a3.

Summarizing, certain antihistamines demonstrate a potential spectrum of antidiabetic properties. Beyond attenuating allergic responses mediated by histamine release, they may prevent histamine-induced hyperglycemia [43,44,45]. By exerting antioxidant and anti-inflammatory effects, these agents may reduce the metabolic demand for glucose and mitigate subsequent hyperglycemic spikes [50,51]. Moreover, they stabilize mast cells, thereby preventing degranulation and the ensuing inflammatory cascades [47,49]. The antagonism of PAF is another notable mechanism that may further attenuate inflammation and associated hyperglycemia [54]. Mechanistically, H1B functions as direct α2-antagonists and inhibitors of ATP-sensitive K^+^ channels, which stimulates insulin secretion from pancreatic β-cells [41,69,70,71,72]. Furthermore, these compounds enhance insulin sensitivity, promoting hormonal and metabolic homeostasis [47,51,54]. Endocrinologically, some antihistamines have also been identified as DPP-4 inhibitors; this inhibition amplifies incretin pathway signalling, thereby ameliorating diabetic dysregulation [52,100]. Collectively, these pleiotropic effects culminate in superior glycemic control and optimized glucose metabolism (Figure 2).

### 4.2. Synergistic Effects of H1 and H2 Receptor Antagonists

As mentioned earlier, histamine exerts its effects through four primary receptors. In addition to H1R, H2R plays a significant role in these signalling pathways. While H2R is primarily known for stimulating gastric acid secretion in parietal cells, it also mediates inflammatory responses, much like H1R [110]. The synergistic effect of co-administering H1 and H2 receptor antagonists is well-documented in conditions characterized by excessive histamine release, such as mastocytosis, where dual blockade effectively alleviates symptoms [111]. Furthermore, this combined therapy has been explored in other clinical contexts [112]. For instance, in COVID-19 patients, dual antihistamine treatment has been shown to reduce systemic inflammation and mitigate disease severity [113]. Similarly, the co-administration of H1R and H2R antagonists has yielded positive outcomes in central nervous system disorders, such as cluster headaches, demonstrating superior efficacy compared to monotherapy [114].

Despite these broad clinical applications, data regarding this synergistic effect on glucose metabolism remain limited. Pini et al. summarized decades of research on the role of histamine in diabetes, highlighting that most therapeutic benefits stem from its anti-inflammatory and metabolism-modulating properties [115]. Other emerging data suggest potential pleiotropic impacts, including, e.g., modulating insulin sensitivity, enhancing the efficacy of oral hypoglycemic agents, and managing diabetic autonomic neuropathy [115,116,117,118]. Nevertheless, elucidating this relationship requires a more mechanistic approach and an in-depth analysis of existing data.

It should also be noted that H2 blockers may possess antiglycation properties. In a recent study, ranitidine was the only H2 blocker to demonstrate significant antiglycation activity. Unlike cimetidine and famotidine, it significantly inhibited BSA glycation in all models, reducing protein carbonyls, glycoxidation markers, and both early and late glycation products. Its effect was comparable to that of aminoguanidine and Trolox and was likely associated with antioxidant activity, including free radical scavenging and metal ion binding [119]. Further research is warranted, particularly in diseases characterized by enhanced protein glycation.

### 4.3. Limitations

Although this systematic review highlights a substantial body of evidence supporting potential antidiabetic effects of antihistamines, these findings should be interpreted carefully. The molecular mechanisms underlying these effects remain not yet fully confirmed. While several studies suggest that H1R blockers may influence glucose metabolism and improve insulin sensitivity through multiple pathways, research directly investigating these mechanisms is limited. Understanding these molecular details is crucial, especially when considering new therapeutic applications. At the same time, pinpointing exact molecular pathways is inherently complex and prone to uncertainty from the very beginning.

Apart from the study by Biedrzycki et al. [57], the available evidence focused on only eight H1 antihistamines, including first-generation agents (antazoline, diphenhydramine, and ketotifen) and second-generation agents (levocetirizine, cetirizine, rupatadine, desloratadine and fexofenadine). Most of the publications reported in vivo studies (n = 12). While these investigations play an essential role in early drug development, their interpretability is limited by considerable methodological diversity. The studies varied widely in drug dosage, duration of experiment, and the animal models employed. Such differences make it challenging to compare outcomes directly and restrict the ability to synthesize the available evidence. Moreover, animal studies inevitably face translational constraints: species-specific differences in metabolism, drug distribution, and receptor expression mean that even promising preclinical findings may not fully translate to human physiology [120]. A similar pattern appears in the clinical trials identified (*n* = 3). Their designs, goals, and reported endpoints differed so markedly that, combined with their small number, they offer only limited support for broader conclusions. The in vitro studies (n = 2) also require cautious interpretation. One [55] relied on a two-dimensional cell culture system, which, despite its practicality, cannot recreate the structural complexity, cell–cell communication, or physiological gradients present in human tissues. These limitations may influence processes such as metabolism, gene expression, and cellular organization [121]. The other study [57] used an acellular bovine serum albumin model, which necessitates higher than physiological compound concentrations and excludes receptor-mediated interactions and homeostatic regulation. Under such simplified conditions, molecules can behave differently than they would in an intact biological environment [122,123]. One study [57] contained in silico analysis over large group of compounds, which gave valuable insight into the potential interactions between candidate drugs and selected molecular targets without the need for time-consuming or costly laboratory experiments. However, this research remains strictly theoretical and cannot fully capture the complexity of biological systems or the environmental conditions that influence pharmacokinetics and pharmacodynamics [124]. Due to the wide heterogeneity of methodologies in terms of implemented substances, dosage regiments, and study models in the reviewed studies, a comprehensive meta-analysis and risk-of-bias assessment were not feasible.

Systematic reviews, including the present one, are valuable tools for synthetizing existing evidence, but they also come with important limitations. Their overall quality is determined largely by the strength and consistency of the studies they incorporate. When available research is rare, heterogeneous, or methodologically limited, as is the case in the topic discussed here, the certainty of the conclusions naturally decreases. Differences in study design, populations, outcome measures, and dosing regimens make direct comparisons challenging and weaken the robustness of any combined assessment. Another well recognized issue is publication bias. Studies reporting negative or inconclusive findings are less likely to reach publication, which may disrupt the overall interpretation of the evidence. Among the 17 studies identified, only 3 presented results, suggesting a lack of antidiabetic effects of H1B. On the one hand, this pattern may appear encouraging, but on the other, it raises the question of whether all unfavourable findings were published. Even comprehensive search strategies cannot fully eliminate this risk. Data extraction and assessment also introduce a degree of subjectivity, despite the use of standardized protocols and independent reviewers. Furthermore, systematic reviews are limited by the state of knowledge at the time of publication, meaning that new evidence may quickly outdate earlier conclusions. Although systematic reviews can highlight trends and gaps, they cannot establish causality and should not be treated as definitive proof without support from high-quality primary studies [125,126].

### 4.4. Next Steps

Although the available evidence is imperfect, it provides a meaningful foundation for further exploration of the antidiabetic properties of antihistamines. H1B constitutes a broad and well-established class of compounds, shaped over decades of clinical use into two major generations. While both reduce H1R activation, they differ substantially in their interactions with other receptors, ability to cross the blood–brain barrier, and, consequently, in their therapeutic profile and adverse-effect spectrum. This systematic review integrates a considerable body of data showing that H1B may influence glucose metabolism and insulin sensitivity. Most of the available studies are in vivo animal experiments, offering valuable insight into how entire organisms respond to these agents under controlled environmental or disease-induced conditions. However, to define the true scope of antihistamines’ metabolic activity, additional steps are needed. A primary task is to clarify the molecular mechanisms responsible for these effects. Several pathways have already been proposed for specific H1-blockers, and some have been partially validated in subsequent in vivo studies. Nonetheless, a systematic effort is required to determine whether these mechanisms are linked to shared structural features across the class or represent drug-specific actions. Computational methods may serve as a useful starting point for identifying previously unrecognized targets, but any in silico findings must be verified in controlled in vitro and in vivo settings to ensure that initial predictions hold in more complex biological systems. Importantly, future studies should investigate more than a single compound at a time and employ standardized experimental parameters, such as dosing strategies, treatment duration, and metabolic endpoints, to enable meaningful comparison across investigations. Only a sufficiently robust and harmonized body of evidence can justify translational efforts, including well-designed clinical trials or considerations of drug repurposing. Equally essential is the transparent reporting of all results, including negative or inconclusive findings. Such completeness is fundamental for building a reliable scientific perspective and for safeguarding future patients as this research field progresses.

## 5. Conclusions

Overall, the available evidence suggests that some histamine H1 receptor antagonists may exert antidiabetic properties. Proposed mechanisms include increased insulin secretion, modulation of immune responses—particularly through the reduction of inflammation—potential nephroprotective effects, and DPP-4 inhibition. Importantly, most of the evidence comes from animal and other preclinical studies; therefore, the applicability of these findings to human physiology and clinical practice remains uncertain. The antihistamines reviewed in this study may warrant further investigation as candidates for drug repurposing in diabetes mellitus. Future research should first establish their molecular targets, effective concentrations, and safety profiles in relevant in vitro models, followed by well-designed animal studies. Translation to human studies should be guided by robust preclinical evidence and involve carefully selected patient populations.

## Figures and Tables

**Table 1:** Examples of 1st- and 2nd-generation antihistamines with their classification and structure.

Name of Compound	Generation	Derivative of	Structure
Antazoline	1st	Ethylenediamine	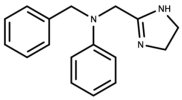
Clemastine	1st	Ethanolamine	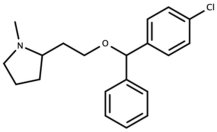
Diphenhydramine	1st	Ethanolamine	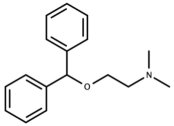
Hydroxizine	1st	Piperazine	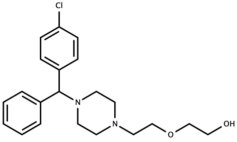
Ketotifen	1st	Benzocycloheptathiophene	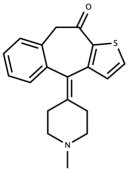
Pheniramine	1st	Alkylamine	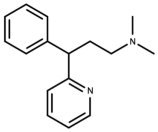
Promethazine	1st	Phenothiazine	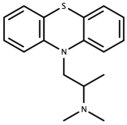
Bilastine	2nd	Piperidine	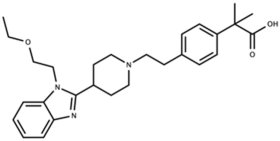
Cetirizine	2nd	Piperazine	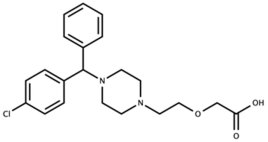
Desloratadine	2nd	Piperidine	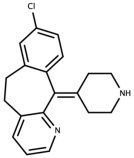
Fexofenadine	2nd	Piperidine	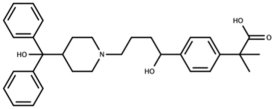
Loratadine	2nd	Piperidine	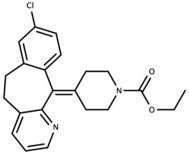
Rupatadine	2nd	Piperidine	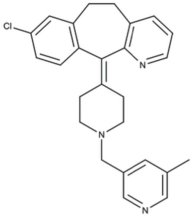

**Table 2:** Inclusion and exclusion criteria in the systematic review.

Inclusion Criteria	Exclusion Criteria
Articles in English	Articles in other languages
Articles describing the antiglycemic properties of diphenhydramine, antazoline, promethazine, ketotifen, clemastine, pheniramine, cetirizine, levocetirizine, bilastine, fexofenadine, desloratadine, loratadine, and rupatadine.	Articles not describing the antiglycemic properties of diphenhydramine, antazoline, promethazine, ketotifen, clemastine, pheniramine, cetirizine, levocetirizine, bilastine, fexofenadine, desloratadine, loratadine and rupatadine.
Research articles (in vitro, ex vivo, in vivo, clinical studies), meta-analyses, systematic literature reviews	Case studies, abstracts

**Table 3:** Characteristics of the included studies. CMC, carboxymethylcellulose; FBG, fasting blood glucose; FPG, fasting plasma glucose; HbA1c, glycaeted haemoglobin; HFD, high fructose diet; HOMA-β, homeostasis model assessment for β-cell function; HOMA-IR, homeostasis model assessment for insulin resistance; JNK, c-Jun N-terminal kinase; MIF, migration inhibitory factor; NOD, non-obese diabetic; OGTT, oral glucose tolerance test; RT-PCR, real-time polymerase chain reaction.

Study Design	Results	Endpoints	References
First-generation H1 antagonists			
Rats intravenously administered antazoline at 1.5 mg/kg and glucose at 0.5 g/kg in the OGTT	During the OGTT, glucose with antazoline induced a greater increase in insulin levels than glucose alone (increase of 139 ± 15 µU/mL vs. 72 ± 18 µU/mL after 2 min).	Antazoline stimulates insulin secretion in vivo and improves glucose tolerance.	Berdeu et al. [41]
Dogs intravenously administered antazoline at 1.5 mg/kg and 0.2 g/kg in OGTT	During the OGTT, glucose with antazoline induced a greater increase in insulin levels than glucose alone (increase of 1062 ± 39 µU/mL vs. 462 ± 183 µU/mL after 2 min).	Antazoline can stimulate insulin secretion in vivo and improve glucose tolerance.	Berdeu et al. [41]
Prediabetic ICA positive patients orally administered ketotifen at 2 mg or 2 × 2 mg/day, for 3 months. Glucose levels were monitored every 4–6 weeks in the OGTT.	Insulin-dependent diabetes with a slow and linear decrease in insulin levels (*p* < 0.05) was observed within 1 year in 7 out of 9 patients. Considerable variations in insulin levels and an increase in glucose concentration were observed in the remaining 2 patients (*p* < 0.05).	Ketotifen was not effective in preventing insulin-dependent diabetes in patients with prediabetes.	Böhmer et al. [42]
Rats intravenously administered diphenhydramine 1 mg/kg and/or metiamide 1 mg/kg with histamine 10 mg/kg after 10 min.	Rats pretreated with diphenhydramine ameliorated increase of blood glucose and reduction of hepatic glycogen compared to control group.	Diphenhydramine prevents hyperglycemic shock.	Dimlich et al. [43]
Rats intravenously administered gatifloxacin 100 mg/kg and diphenhydramine 1 mg/kg 5 min earlier. Blood samples were collected 0, 5, 15, 30, and 60 min after gatifloxacin injection to measure glycaemia.	Diphenhydramine pretreatment prevented glycemia increase caused by gatifloxacin in every assessment (*p* < 0.05).	Diphenhydramine reduces gatifloxacin-induced hyperglycaemic.	Ishiwata et al. [44]
Rats intravenously administered levofloxacin 300 mg/kg and diphenhydramine 1 mg/kg 5 min earlier intravenously. Blood samples were collected 0, 5, 15, 30, and 60 min after levofloxacin injection to measure glycaemia.	Diphenhydramine pretreatment prevented glycemia increase caused by levofloxacin in every assessment (*p* < 0.05).	Diphenhydramine reduces levofloxacin-induced hyperglycaemia.	Ishitawa et al. [45]
Rats administered talc 10 mg/kg orally for 21 days and/or diphenhydramine 1 mg/kg intravenously (after 21 days, if talc was administered).	Diphenhydramine injection resulted in significant (*p* < 0.0001) decrease of blood glucose level.	Diphenhydramine restores glucose metabolism	Afzal et al. [46]
Streptozocin-induced diabetic rats administered 0.09 mg/kg/d of ketotifen intragastrically. Animals were observed for 8 weeks, with measurements taken in weeks 0, 4, and 8.	Ketotifen group, compared to diabetes group, was observed to significantly reduce fasting plasma glucose (*p* < 0.05), fasting insulin (*p* < 0.05), and HOMA-IR (*p* < 0.05) after 4 and 8 weeks of the experiment’s duration.	Ketotifen improves impaired glucose metabolism and insulin resistance.	Chen et al. [47]
Patients with BMI ≥ 30 kg/m^2^ and type 2 DM administered glimepiride 3 mg/day and/or ketotifen 1 mg twice a day. The experiment lasted 12 weeks.	The group treated with glimepiride + ketotifen twice a day had significantly decreased level of FBG (*p* < 0.01) and Hb1Ac (*p* < 0.01) compared to glimepiride only.	Ketotifen mends glucose metabolism.	El-Haggar et al. [48]
WT *Kit^W-sh/W-sh^* and *Kit^WvWv^* mice fed with Western diet, then continued or switched to a chow diet and administered ketotifen 25 mg/kg/day intraperitoneally. Glucose tolerance was measured by the end of each course of Western diet consumption and 8 weeks of ketotifen treatment.	Ketotifen administration in WT mice fed with Western diet improved glucose tolerance (*p* < 0.05) to similar levels as *Kit^W-sh/W-sh^* and *Kit^WvWv^* mice with or without treatment.	Ketotifen attenuates glucose metabolism disorders.	Liu et al. [49]
Second-generation H1 antagonists			
Streptozotocin-induced diabetic rats administered orally: 0.5% CMC 3 mL/kg BW/day or losartan 25 mg/kg/d in 0.5% CMC or levocetirizine 0.5 mg/kg BW/day in 0.5% CMC for 8 weeks.	Glycemia was more effectively controlled with levocetirizine than losartan. HbA1c values were restored to normal levels in levocetirizine-treated rats relative to the control group (*p* > 0.05), diabetes groups, and losartan-treated diabetes groups (*p* < 0.05).	Levocetirizine promotes glycemia control.	Anbar et al. [50]
Rats fed with HFD administered 0.5% CMC 3 mL/kg BW or pioglitazone 5 mg/kg BW/day or levocetirizine 0.5 mg/kg BW/day).	Levocetirizine and pioglitazone decreased FBG levels to the values noted in the control group (*p* < 0.05). Fasting insulin levels were higher in HFD groups than the control group, but the difference was not significant (95% CI = 0.11–0.51, *p* < 0.05).	Levocetirizine decreases insulin resistance and improves glucose tolerance.	Shawky et al. [51]
Patients administered ramipril and/or fexofenadine 60 mg dailyfor 6 months. FBG and HbA1c in blood samples were measured in the beginning of the study and after 6 months.	Fexofenadine group revealed significant decrease in level of FBG and HbA1c compared to control group, which exhibited increase in levels of these biomarkers (*p* = 0.003 for FBG and *p* = 0.001 for HbA1c).	Fexofenadine minimizes FBG and HbA1c levels.	El-fatary et al. [52]
Rats administered desloratadine 50 μg/kg/day. Blood glucose and insulin level were measured after 15 weeks of the experiment.	Experimental group exhibited significantly higher level of glycemia (*p* < 0.05) and non-fasting insulin (*p* < 0.05).	Prolonged desloratadine administration impairs glucose metabolism and insulin sensitivity.	Gasheva et al. [53]
Rats divided into five groups: control, rupatadine 6 mg/kg/day, diabetic (induced with 50 mg/kg streptozocin), diabetic + rupatadine 3 mg/kg/day, diabetic + rupatadine 6 mg/kg/day. The experiment took 4 weeks and, at the end, blood glucose and insulin levels were assessed.	Diabetic + rupatadine (3 and 6 mg/kg/day) groups were observed to significantly decrease blood glucose levels (*p* < 0.05) and increase insulin concentration (*p* > 0.05), compared to diabetic group.	Rupatadine mitigates glucose level growth and insulin level reduction.	Hafez et al. [54]
Insulin-producing mouse β-TC6 cells treated with either IL-1β 50 U/mL with IFN-γ 1000 U/mL or H**_2_**O**_2_** 125 μM. Then subdivided into three groups depending on the H1-receptor antagonist used: cetirizine (5 nM, 20 nM, 50 nM, and 200 nM), desloratadine (10 nM, 50 nM, 100 nM, and 500 nM) and vehicle only. After 24 h of exposure, propidium iodide stain was used to depict apoptotic and necrotic cells. MIF, JNK, and β-catenin levels were assessed using immunoblotting methods. c-Jun expression was established using RT-PCR.	5 nM and 20 nM cetirizine significantly prevented β-TC6 cells from cytokine-induced cell death by approx. 20%. 20 nM cetirizine significantly lowered H_2_O_2_-induced cell death (*p* = 0.024). 20 nM cetirizine and 50 desloratadine mitigated cytokine-induced MIF level decrease (*p* = 0.041). 20 nM cetirizine counteracted JNK phosphorylation (*p* = 0.032) and c-Jun expression (*p* = 0.042). Both cetirizine and desloratadine boosted β-catenin levels (*p* < 0.05).	Cetirizine especially prevents β-TC6 cells from cytokine and H_2_O_2_-induced death and impaired functioning.	Anvari et al. [55]
Mice administered cetirizine 25 mg/kg/day. Every week, their blood samples were tested weekly to evaluate hyperglycaemia appearance and diabetes development.	Cetirizine was not effective in preventing diabetes development (*p* > 0.05) in female NOD mice.	Cetirizine did not restore insulin sensitivity or prevent diabetes development.	Anvari et al. [56]
Mice fed with HFD and administered cetirizine 25 mg/kg/day. Blood samples were collected before cetirizine administration and after each week of two-week treatment to evaluate changes in glycemia, glucose fasting, and insulin sensitivity.	One-week cetirizine treatment allowed mice to normalize glycemia in HFD group, with no significant difference vs. control group (*p* > 0.05). Two-week cetirizine therapy resulted in significantly better glucose tolerance in HFD-fed mice compared to control group (*p* < 0.05), but not insulin sensitivity (*p* > 0.05).	Cetirizine is efficient in regulation of blood glucose levels and improving glucose fasting.	Anvari et al. [56]
First- and second-generation H1 antagonists			
First- (diphenhydramine, antazoline, promethazine, ketotifen, clemastine, pheniramine) and second- (cetirizine, levocetirizine, bilastine, fexofenadine, loratadine, desloratadine) generation antihistamines were used in an in vitro experimental model of protein glycoxidation with bovine serum albumin and glucose, fructose, ribose, galactose, glyoxal, and methylglyoxal as glycation agents. The measured parameters of protein glycation were: Amadori products, β-amyloid, and advanced glycation end products.	Majority of the examined compounds did not significantly decrease protein glycation parameters in an in vitro study.	Both first- and second-generation antihistamines did not represent antiglycation potential.	Biedrzycki et al. [57]

## Data Availability

No new data were created or analyzed in this study. Data sharing is not applicable to this article.

## Supplementary Materials

The following supporting information can be downloaded at: https://www.mdpi.com/article/10.3390/antiox15091098/s1, Table S1. Outcome of quality assessment of retrieved clinical trial using the National Heart, Lung, and Blood Institute (NHLBI) Quality Assessment Tools. ELISA, Enzyme-Linked Immunosorbent Assay; FPIR, first-phase insulin response; HbA1c, glycated haemoglobin; ICAs, islet cell antibodies; IVGTT, intravenous glucose tolerance test; Table S2. Outcome of quality assessment of retrieved animal studies using the Systematic Review Centre for Laboratory animal Experimentation (SYRCLE) Risk of Bias tool; Table S3. Outcome of quality assessment of retrieved in vitro studies using the QUIN (Quality Assessment Tool For In Vitro Studies) tool.

## Abbreviations

The following abbreviations are used in this manuscript:

AGEs	advanced glycation end products
BSA	bovine serum albumin
cAMP	cyclic adenosine monophosphate
CRP	C-reactive protein
CMC	carboxymethylcellulose
DAO	diamine oxidase
DM	diabetes mellitus
DPP-4	dipeptidyl peptidase-4
ECL	extracellular loop
ELISA	Enzyme-Linked Immunosorbent Assay
FIPR	first-phase insulin response
FBG	fasting blood glucose
GFR	glomerular filtration rate
GLP-1	glucagon-like peptide-1
GPCRs	G protein-coupled receptors
GSH	reduced glutathione
H1B	H1 blockers
H1R	H1 receptor
H2R	H2 receptor
HbA1c	glycated haemoglobin
HDL-C	high-density lipoprotein-cholesterol
HNMT	histamine N-methyltransferase
HOMA-β	homeostasis model assessment of β-cell function
ICL	intracellular loop
IFN-γ	interferon-γ
IL-6	interleukin-6
ICAs	islet cell antibodies
IVGTT	intravenous glucose tolerance test
LTC4	leukotriene C4
MAO-B	monoamine oxidase-B
MDA	malondialdehyde
NADPH	nicotinamide adenine dinucleotide phosphate
NEPG	non-enzymatic protein glycation
NF-κB	nuclear factor kappa B
NOS	nitric oxide synthase
OGTT	oral glucose tolerance test
PAF	platelet-activating factor
PLC	phospholipase C
RAGE	receptor for advanced glycation end products
RNS	reactive nitrogen species
ROS	reactive oxygen species
SOD	superoxide dismutase
TGF-α	transforming growth factor α
TGF-β	transforming growth factor beta-1
TM	transmembrane
TNF-α	tumour necrosis factor alpha
WT	wild type

## Appendix A

Appendix A contains the list of keywords used in the process of literature search.

Keywords used in literature search for this review: (diphenhydramine + diabetes, antazoline + diabetes, promethazine + diabetes, ketotifen + diabetes, clemastine + diabetes, pheniramine + diabetes, cetirizine + diabetes, levocetirizine + diabetes, bilastine + diabetes, fexofenadine + diabetes, desloratadine + diabetes, loratadine + diabetes, rupatadine + diabetes), (diphenhydramine + insulin resistance, antazoline + insulin resistance, promethazine + insulin resistance, ketotifen + insulin resistance, clemastine + insulin resistance, pheniramine + insulin resistance, cetirizine + insulin resistance, levocetirizine + insulin resistance, bilastine + insulin resistance, fexofenadine + insulin resistance, desloratadine + insulin resistance, loratadine + insulin resistance, rupatadine + insulin resistance), (diphenhydramine + glycation, antazoline + glycation, promethazine + glycation, ketotifen + glycation, clemastine + glycation, pheniramine + glycation, cetirizine + glycation, levocetirizine + glycation, bilastine + glycation, fexofenadine + glycation, desloratadine + glycation, loratadine + glycation, rupatadine + glycation), (diphenhydramine + antiglycation, antazoline + antiglycation, promethazine + antiglycation, ketotifen + antiglycation, clemastine + antiglycation, pheniramine + antiglycation, cetirizine + antiglycation, levocetirizine + antiglycation, bilastine + glycation, fexofenadine + glycation, desloratadine + glycation, loratadine + antiglycation, rupatadine + antiglycation), (diphenhydramine + carbonyl stress, antazoline + carbonyl stress, promethazine + carbonyl stress, ketotifen + carbonyl stress, clemastine + carbonyl stress, pheniramine + carbonyl stress, cetirizine + carbonyl stress, levocetirizine + carbonyl stress, bilastine + carbonyl stress, fexofenadine + carbonyl stress, desloratadine + carbonyl stress, loratadine + carbonyl stress, rupatadine + carbonyl stress), (diphenhydramine + AGEs, antazoline + AGEs, promethazine + AGEs, ketotifen + AGEs, clemastine + AGEs, pheniramine + AGEs, cetirizine + AGEs, levocetirizine + AGEs, bilastine + AGEs, fexofenadine + AGEs, desloratadine + AGEs, loratadine + AGEs, rupatadine + AGEs), (diphenhydramine + glycemia, antazoline + glycemia, promethazine + glycemia, ketotifen + glycemia, clemastine + glycemia, pheniramine + glycemia, cetirizine + glycemia, levocetirizine + glycemia, bilastine + glycemia, fexofenadine + glycemia, desloratadine + glycemia, loratadine + glycemia, rupatadine + glycemia), (diphenhydramine + hyperglycaemia, antazoline + hyperglycaemia, promethazine + hyperglycaemia, ketotifen + hyperglycaemia, clemastine + hyperglycaemia, pheniramine + hyperglycaemia, cetirizine + hyperglycaemia, levocetirizine + hyperglycaemia, bilastine + hyperglycaemia, fexofenadine + hyperglycaemia, desloratadine + hyperglycaemia, loratadine + hyperglycaemia, rupatadine + hyperglycaemia).
