# Supplementary material for: Antidiabetic Properties of Histamine H1 Receptor Antagonists: A Systematic Review
Source: Antioxidants (Basel). 2026 Aug 31;15(9):1098. doi: 10.3390/antiox15091098 (PMC13603188; doi:10.3390/antiox15091098)
Supplement: Supplementary file 1 [file antioxidants-15-01098-s001.zip › Table S3.pdf]

Table S3. Outcome of quality assessment of retrieved in vitro studies using the QUIN (Quality Assessment Tool For In Vitro Studies) tool.

	QUIN Tool Criteria	Biedrzycki et al. (2024) [1]	Anvari et al. (2014) [2]
1	Clearly stated aims/objectives	2	2
2	Detailed explanation of sample size calculation	0	0
3	Detailed explanation of sampling technique	N/A	N/A
4	Details of comparison group	2	2
5	Detailed explanation of methodology	2	2
6	Operator details	0	0
7	Randomization	0	0
8	Method of measurement of outcome	2	2
9	Outcome assessor details	0	0
10	Blinding	0	0
11	Statistical analysis	2	1
12	Presentation of results	2	2
	**Total Score**	**12/22**	**11/12**
	**Final Percentage**	**54.5%**	**50.0%**
	**Overall Risk of Bias**	**Medium**	**Medium**

References:

1. Biedrzycki, G.; Wolszczak – Biedrzycka, B.; Dorf, J.; Michalak, D.; Żendzian – Piotrowska, M.; Zalewska, A.; Maciejczyk, M. The Antiglycation Potential of H1 Receptor Antagonists – in Vitro Studies in Bovine Serum Albumin Model and in Silico Molecular Docking Analyses. *Biomedicine & Pharmacotherapy* **2024**, *175*, 116632, doi:10.1016/j.biopha.2024.116632.
2. Anvari, E.; Fred, R.G.; Welsh, N. The H1-Receptor Antagonist Cetirizine Protects Partially Against Cytokine- and Hydrogen Peroxide–Induced β-TC6 Cell Death In Vitro. *Pancreas* **2014**, *43*, 624–629, doi:10.1097/MPA.0000000000000076.
