# Supplementary material for: Antidiabetic Properties of Histamine H1 Receptor Antagonists: A Systematic Review
Source: Antioxidants (Basel). 2026 Aug 31;15(9):1098. doi: 10.3390/antiox15091098 (PMC13603188; doi:10.3390/antiox15091098)
Supplement: Supplementary file 1 [file antioxidants-15-01098-s001.zip › Table S1.pdf]

Table S1. Outcome of quality assessment of retrieved clinical trial using the National Heart, Lung, and Blood Institute (NHLBI) Quality Assessment Tools. ELISA, Enzyme-Linked Immunosorbent Assay; FPIR, first-phase insulin response; HbA1c, glycated haemoglobin; ICAs, islet cell antibodies; IVGTT, intravenous glucose tolerance test

		El-fatatry et al., 2023 [1]		El-Haggar et al., 2015 [2]		Böhmer et al., 1994 [3]	
	**NHLBI Question**	**Rating**	**Justification**	**Rating**	**Justification**	**Rating**	**Justification**
1	**Was the study described as randomized, a randomized trial, a randomized clinical trial, or an RCT?**	Yes	Patients were randomized into two groups.	Yes	The study design was a parallel randomized controlled study.	Yes	It was a randomized double-blind placebo-controlled study.
2	**Was the method of randomization adequate (i.e., use of randomly generated assignment)?**	No	A simple randomization method based on hospital visit days was used. This is quasi-randomization (predictable sequence), which is considered inadequate in EBM.	NR	The text does not explain how the randomization sequence was generated.	NR	The method of random sequence generation is not detailed.
3	**Was the treatment allocation concealed (so that assignments could not be predicted)?**	NR	No mention of sealed envelopes or centralized systems.	NR	No information is provided in the text.	NR	No specifics on allocation concealment are mentioned.
4	**Were study participants and providers blinded to treatment group assignment?**	No	The study was an open-labeled randomized controlled clinical study.	NR	There is no mention of blinding in the methodology.	Yes	The medication was administered in a double-blinded fashion.
5	**Were the people assessing the outcomes blinded to the participants' group assignments?**	NR	No information is provided in the text regarding this.	NR	No information is provided.	Yes	This is implicitly covered by the rigorous double-blind protocol.
6	**Were the groups similar at baseline on important characteristics**	Yes	Baseline demographic and clinical characteristics	Yes	There were no significant differences in demographic or	CD	Due to the very small sample size (9 patients total) and varying baseline

	**that could affect outcomes (e.g., demographics, risk factors, co-morbid conditions)?**		were generally comparable in both groups.		anthropometric parameters between the groups at baseline.		characteristics, a meaningful baseline comparison between groups cannot be determined.
7	**Was the overall drop-out rate from the study at endpoint 20% or lower of the number allocated to treatment?**	Yes	Out of 76 enrolled patients , 15 were lost to follow-up, resulting in a drop-out rate of approximately 19.7%.	Yes	48 patients were enrolled and divided into three groups of 16. Data is presented for all 16 patients per group at 12 weeks, implying a 0% drop-out rate.	Yes	Results and individual data are presented for all 9 patients initially enrolled. Study terminations were due to clinical endpoints (e.g., reaching diabetes criteria), not loss to follow-up.
8	**Was the differential drop-out rate (between treatment groups) at endpoint 15 percentage points or lower?**	Yes	Eight patients in the control group and seven in the fexofenadine group were lost. The difference is minimal.	NA	Not applicable due to 0% drop-out.	NA	No loss to follow-up was reported.
9	**Was there high adherence to the intervention protocols for each treatment group?**	NR	Patients had regular monthly visits for medication refills, but the exact compliance percentage is not reported.	NR	Patients were followed up for compliance, but specific adherence rates are not reported.	Yes	The medication did not have to be interrupted in any of the subjects.
10	**Were other interventions avoided or similar in the groups (e.g., similar background treatments)?**	Yes	Medications were reviewed monthly to exclude interacting drugs , and no major changes were made to their diabetic regimen.	Yes	Medical history was taken to ensure the absence of interacting or interfering drugs.	NR	No mention of monitoring diet, lifestyle, or concurrent therapies.
11	**Were outcomes assessed using valid and reliable measures, implemented consistently across all study participants?**	Yes	Urine and blood samples were collected at baseline and after six months using standard ELISA kits and colorimetric methods.	Yes	Blood samples were tested using standard colorimetric and ELISA methods across all groups.	Yes	Patients were consistently followed with sequential IVGTTs and standard assays.
12	**Did the authors report that the sample size was sufficiently large**	Yes	The authors calculated that 30 participants were needed in each	NR	There is no mention of sample size or	No	The authors admit low power, stating it is "most unlikely that a clinically significant

	**to be able to detect a difference in the main outcome between groups with at least 80% power?**		arm to achieve 80% power.		power calculations.		difference... would have been detected, even with large numbers".
13	**Were outcomes reported or subgroups analyzed prespecified (i.e., identified before analyses were conducted)?**	Yes	The study was prospectively registered (NCT04224428) , and primary and secondary endpoints were clearly defined.	NR	No clinical trial registration number or pre-published protocol is referenced.	Yes	FPIR, HbA1c, ICAs, and insulin autoantibodies were explicitly tracked according to the study design.
14	**Were all randomized participants analyzed in the group to which they were originally assigned, i.e., did they use an intention-to-treat analysis?**	No	Only the 61 patients who completed the study were included in the per-protocol analysis.	Yes	All initially assigned participants appear to have been analyzed in their respective groups.	Yes	Individual data (including graphs and tables) are provided for every single assigned subject.
	**Overall Rating**		**Fair**		**Fair**		**Fair**

References:

1. El-fatatry, B.M.; El-Haggar, S.M.; Ibrahim, O.M.; Shalaby, K.H. Repurposing Fexofenadine as a Promising Candidate for Diabetic Kidney Disease: Randomized Clinical Trial. *Int. Urol. Nephrol.* **2023**, *56*, 1395–1402, doi:10.1007/s11255-023-03804-w.
2. El-Haggar, S.M.; Farrag, W.F.; Kotkata, F.A. Effect of Ketotifen in Obese Patients with Type 2 Diabetes Mellitus. *J. Diabetes Complications* **2015**, *29*, 427–432, doi:10.1016/j.jdiacomp.2015.01.013.
3. Böhmer, K.P.; Kolb, H.; Kuglin, B.; Zielasek, J.; Hübinger, A.; Lampeter, E.F.; Weber, B.; Kolb-Bachofen, V.; Jastram, H.U.; Bertrams, J.; et al. Linear Loss of Insulin Secretory Capacity During the Last Six Months Preceding IDDM: No Effect of Antiedematous Therapy with Ketotifen. *Diabetes Care* **1994**, *17*, 138–141, doi:10.2337/diacare.17.2.138.
