# Supplementary material for: Antidiabetic Properties of Histamine H1 Receptor Antagonists: A Systematic Review
Source: Antioxidants (Basel). 2026 Aug 31;15(9):1098. doi: 10.3390/antiox15091098 (PMC13603188; doi:10.3390/antiox15091098)
Supplement: Supplementary file 1 [file antioxidants-15-01098-s001.zip › Table S2.pdf]

Table S2. Outcome of quality assessment of retrieved animal studies using the Systematic Review Centre for Laboratory animal Experimentation (SYRCLE) Risk of Bias tool.

	SYRCLE Question	Chen et al. (2017) [1]	Afzal et al. (2018) [2]	Gasheva et al. (2019) [3]	Ishiwata et al. (2006) [4]	Ishiwata et al. (2010) [5]	Liu et al. (2009) [6]	Dimlich (1985) [7]	Hafez et al. (2020) [8]	Anvari et al. (2015) [9]	Shawky et al. (2014) [10]	Anbar et al. (2016) [11]	Berdeu et al. (1997) [12]
1.	**Was the allocation sequence adequately generated and applied?**	Unclear	Unclear	Unclear	Unclear	Unclear	Unclear	Unclear	Unclear	Unclear	Unclear	Unclear	Unclear
2.	**Were the groups similar at baseline or were they adjusted for confounders in the analysis?**	Yes	Yes	Yes	Yes	Yes	Yes	Yes	Yes	Yes	Yes	Yes	Yes
3.	**Was the allocation adequately concealed?**	Unclear	Unclear	Unclear	Unclear	Unclear	Unclear	Unclear	Unclear	Unclear	Unclear	Unclear	Unclear
4.	**Were the animals randomly housed during the experiment?**	Unclear	Unclear	Unclear	Unclear	Unclear	Unclear	Unclear	Unclear	Unclear	Unclear	Unclear	Unclear
5.	**Were the caregivers and/or investigators blinded from knowledge which intervention each animal received during the experiment?**	Unclear	Unclear	Unclear	Unclear	Unclear	Unclear	Unclear	Unclear	Unclear	Unclear	Unclear	Unclear
6.	**Were animals selected at random for outcome assessment?**	Unclear	Unclear	Unclear	Unclear	Unclear	Unclear	Unclear	Unclear	Unclear	Unclear	Unclear	Unclear
7.	**Was the outcome assessor blinded?**	Unclear	Unclear	Unclear	Unclear	Unclear	Yes / Unclear	Unclear	Unclear	Unclear	Yes / Unclear	Unclear	Unclear
8.	**Were incomplete outcome data adequately addressed?**	Yes	Yes	Yes	Yes	Yes	Yes	Yes	Yes	Yes	Yes	Yes	Yes
9.	**Are reports of the study free of selective outcome reporting?**	Yes	Yes	Yes	Yes	Yes	Yes	Yes	Yes	Yes	Yes	Yes	Yes
10.	**Was the study apparently free of other problems that could result in high risk of bias?**	Yes	Yes	Yes	Yes	Yes	Yes	Yes	Yes	Yes	Yes	Yes	Yes

References:

1. Chen, Z.; Sun, H.; Wang, J.; Ni, L.; Gu, X.; Ge, S.; Chen, Q.; Mu, L.; Cheng, X. Role of Ketotifen on Metabolic Profiles, Inflammation and Oxidative Stress in Diabetic Rats. *Endocr. J.* **2017**, *64*, 411–416, doi:10.1507/endocrj.EJ16-0458.
2. Afzal, M.; Saleem, S.; Singh, N.; Kazmi, I.; Khan, R.; Nadeem, M.S.; Zamzami, M.A.; Al-Abbasi, F.A.; Anwar, F. Evaluation of Diphenhydramine in Talc Induced Type 2 Diabetes Mellitus in Wistar Rats. *Biomedicine & Pharmacotherapy* **2018**, *97*, 652–655, doi:10.1016/j.biopha.2017.10.085.

3. Gasheva, O.Y.; Tsoy Nizamutdinova, I.; Hargrove, L.; Gobbell, C.; Troyanova-Wood, M.; Alpini, S.F.; Pal, S.; Du, C.; Hitt, A.R.; Yakovlev, V. V.; et al. Prolonged Intake of Desloratadine: Mesenteric Lymphatic Vessel Dysfunction and Development of Obesity/Metabolic Syndrome. *American Journal of Physiology-Gastrointestinal and Liver Physiology* **2019**, *316*, G217–G227, doi:10.1152/ajpgi.00321.2018.
4. Ishiwata, Y.; Itoga, Y.; Yasuhara, M. Effect of Levofloxacin on Serum Glucose Concentration in Rats. *Eur. J. Pharmacol.* **2006**, *551*, 168–174, doi:10.1016/J.EJPHAR.2006.08.081.
5. Ishiwata, Y.; Yasuhara, M. Gatifloxacin-Induced Histamine Release and Hyperglycemia in Rats. *Eur. J. Pharmacol.* **2010**, *645*, 192–197, doi:10.1016/J.EJPHAR.2010.07.032.
6. Liu, J.; Divoux, A.; Sun, J.; Zhang, J.; Clément, K.; Glickman, J.N.; Sukhova, G.K.; Wolters, P.J.; Du, J.; Gorgun, C.Z.; et al. Genetic Deficiency and Pharmacological Stabilization of Mast Cells Reduce Diet-Induced Obesity and Diabetes in Mice. *Nat. Med.* **2009**, *15*, 940–945, doi:10.1038/nm.1994.
7. Dimlich, R.V. Histamine Antagonists in the Treatment of Shock Hyperglycemia in the Rat. *Ann. Emerg. Med.* **1985**, *14*, 91–96, doi:10.1016/S0196-0644(85)81067-2.
8. Hafez, H.M.; Abdel-Hakeem, E.A.; Hassanein, H. Rupatadine, a Dual Antagonist of Histamine and Platelet-Activating Factor (PAF), Attenuates Experimentally Induced Diabetic Nephropathy in Rats. *Naunyn. Schmiedebergs. Arch. Pharmacol.* **2020**, *393*, 1487–1500, doi:10.1007/s00210-020-01856-8.
9. Anvari, E.; Wang, X.; Sandler, S.; Welsh, N. The H1-Receptor Antagonist Cetirizine Ameliorates High-Fat Diet-Induced Glucose Intolerance in Male C57BL/6 Mice, but Not Diabetes Outcome in Female Non-Obese Diabetic (NOD) Mice. *Ups. J. Med. Sci.* **2015**, *120*, 40–46, doi:10.3109/03009734.2014.967422.
10. Shawky, N.M.; Shehatou, G.S.G.; Abdel Rahim, M.; Suddek, G.M.; Gameil, N.M. Levocetirizine Ameliorates High Fructose Diet-Induced Insulin Resistance, Vascular Dysfunction and Hepatic Steatosis in Rats. *Eur. J. Pharmacol.* **2014**, *740*, 353–363, doi:10.1016/j.ejphar.2014.07.021.
11. Anbar, H.S.; Shehatou, G.S.G.; Suddek, G.M.; Gameil, N.M. Comparison of the Effects of Levocetirizine and Losartan on Diabetic Nephropathy and Vascular Dysfunction in Streptozotocin-Induced Diabetic Rats. *Eur. J. Pharmacol.* **2016**, *780*, 82–92, doi:10.1016/j.ejphar.2016.03.035.
12. Berdeu, D.; Puech, R.; Ribes, G.; Loubatières-Mariani, M.-M.; Bertrand, G. Antazoline Increases Insulin Secretion and Improves Glucose Tolerance in Rats and Dogs. *Eur. J. Pharmacol.* **1997**, *324*, 233–239, doi:10.1016/S0014-2999(97)00126-X.
